# A new method for the in vivo identification of degenerated material property ranges of the human eye: feasibility analysis based on synthetic data

**DOI:** 10.1007/s10237-021-01541-6

**Published:** 2021-12-20

**Authors:** Stefan Muench, Mike Roellig, Daniel Balzani

**Affiliations:** 1 Department of Testing of Electronics and Optical Methods, Fraunhofer Institute for Ceramic Technologies and Systems IKTS, Dresden, Germany; 2 Chair of Continuum Mechanics, Ruhr University Bochum, Bochum, Germany

**Keywords:** Inverse problems, Keratoconus, Unique identification, Cornea, Non-contact tonometer

## Abstract

This paper proposes a new method for in vivo and almost real-time identification of biomechanical properties of the human cornea based on non-contact tonometer data. Further goal is to demonstrate the method’s functionality based on synthetic data serving as reference. For this purpose, a finite element model of the human eye is constructed to synthetically generate full-field displacements from different data sets with keratoconus-like degradations. Then, a new approach based on the equilibrium gap method combined with a mechanical morphing approach is proposed and used to identify the material parameters from virtual test data sets. In a further step, random absolute noise is added to the virtual test data to investigate the sensitivity of the new approach to noise. As a result, the proposed method shows a relevant accuracy in identifying material parameters based on full-field displacements. At the same time, the method turns out to work almost in real time (order of a few minutes on a regular workstation) and is thus much faster than inverse problems solved by typical forward approaches. On the other hand, the method shows a noticeable sensitivity to rather small noise amplitudes rendering the method not accurate enough for the precise identification of individual parameter values. However, analysis show that the accuracy is sufficient for the identification of property ranges which might be related to diseased tissues. Thereby, the proposed approach turns out promising with view to diagnostic purposes.

## Introduction

Eye diseases are particularly harmful for those affected. They impair vision and thus separate people from their environment and society. In particular, children and adolescents are handicapped in their individual development by eye diseases. A typical eye disease that occurs during puberty is keratoconus (Kennedy et al. 1986; Krachmer et al. 1984; Raiskup et al. 2016). The disease results in softening and deformation of the transparent anterior part of the outer shell of the eye, called cornea, under intraocular pressure (IOP) (Krachmer et al. 1984; Raiskup et al. 2016; Bron 1988). This leads to defective vision. A fast, early and reliable diagnosis of eye diseases such as keratoconus is essential for early treatment. The objective is to keep the impact on vision to a minimum. Measuring the biomechanical properties of the cornea in vivo is promising for this purpose. So far, they cannot be measured nondestructively. On the contrary, the lack of knowledge about the biomechanical properties and their neglect in the measurement of other quantities, such as the IOP, leads to measurement deviations and incorrect estimations of the IOP (Liu and Roberts 2005).

Air pulse-based non-contact tonometers (NCT) such as the Ocular Response Analyzer (ORA) from Reichert Inc. (Buffalo, USA) and the Corvis® ST from Oculus Optikgeräte GmbH (Wetzlar, Germany) are widely used as standard methods for intraocular pressure measurement. Especially during the measurement with the Corvis® ST, large amounts of load–displacement data are generated, which have not been fully utilized so far. Currently, two stiffness parameters SP-A1 and SP-HC exist, which indicate the resistance of the cornea to deformation. They are calculated from pressures determined by the device at specific times with respect to the indentation depth of the cornea (Roberts et al. 2017; Vinciguerra et al. 2016). In addition, the Corvis biomechanical index (CBI) is used. It is based on a nonlinear function of various Corvis® ST parameters determined via a regression analysis to separate healthy eyes from eyes suffering from keratoconus (Vinciguerra et al. 2016). Another new approach is the stress–strain index (SSI). It reduces the comparison of a measured stress–strain curve with a reference curve for healthy corneas to a scalar value. The reference curve is based on experimental data (Eliasy et al. 2019). The mentioned quantities are based on purely empirical functions, so that they are only applicable in defined ranges. For example, the SSI is only valid for corneas with normal topography, which already excludes keratoconic eyes (Eliasy et al. 2019). Furthermore, the parameters do not provide structural information of the corneal tissue. However, the available data provide the basis for linking biomechanical properties of the cornea with structural material properties such as collagen fiber stiffness. Such parameters obtained in vivo would be a key to earlier and more accurate diagnosis of many other eye diseases. Consequently, there are already publications to use the experimental basis from the NCT for the inverse identification of biomechanical material properties using finite elements (FEs) (Elsheikh et al. 2014; Whitford et al. 2015). In this process, associated mechanical fields inside the cornea are calculated based on specified deformations or forces and subsequently compared with the experimental data. However, these so-called finite element update methods (FEMU) are computationally expensive, and so, it is very difficult to raise them out of the laboratory stage. Therefore, in the following we would like to propose a method that allows both a determination of structural material properties and to be update-free and thus almost real-time capable, so that it can be implemented directly in the medical examination device. The method is based on the equilibrium gap method (EGM), see, e.g., (Avril et al. 2008; Amiot et al. 2012), and a special treatment of different parameters allowing for a quadratic relation between the remaining material parameters and the resulting objective function for the parameter identification. Major disadvantage of the proposed method is that the full-field reconstructions of the displacement fields are required as input which are not yet fully available in 3D on standard medical devices. This makes the method potentially sensitive to noise and to deviations from incompressibility, which will be shown in the analysis section. Since currently not all necessary input variables can be acquired by the Corvis® ST, an approach for data enrichment is presented, which forms the link between a realistic measurement and the presented inverse approach. This approach allows for a construction of 3D displacement data from the currently available 2D images and ensures incompressibility in the input kinematics data.

## Materials and methods

For the investigation of the method, synthetic data obtained from FE simulations of the NCT are used as a database. The advantage of this approach is that reference data sets with uniquely assigned material properties can be generated for test purposes. Thus, the accuracy of the presented approaches can be quantified and investigations regarding the influence of image noise on the determined characteristic values can be carried out. The FE simulations were performed with the software ANSYS® Mechanical APDL, Release 18.2. The model of the eye is based on a detailed geometry with a representation of the internal structure of the eye. It uses hydrostatic fluid elements to represent the IOP including its change during NCT and uses an iterative procedure to calculate the stress-free geometry. The model uses well-considered boundary conditions and a cornea-specific material model to represent the anisotropic and hyperelastic behavior of the cornea. Both the geometry and material models are fed by properties based on a literature review. Thus, it can be considered as a characteristic and state-of-the-art model of the human eye, which is underlined by the good agreement between the simulated deformation behavior and the deformation behavior of a cornea during NCT published in the literature.

### Virtual non-contact tonometry

**Fig. 1:**
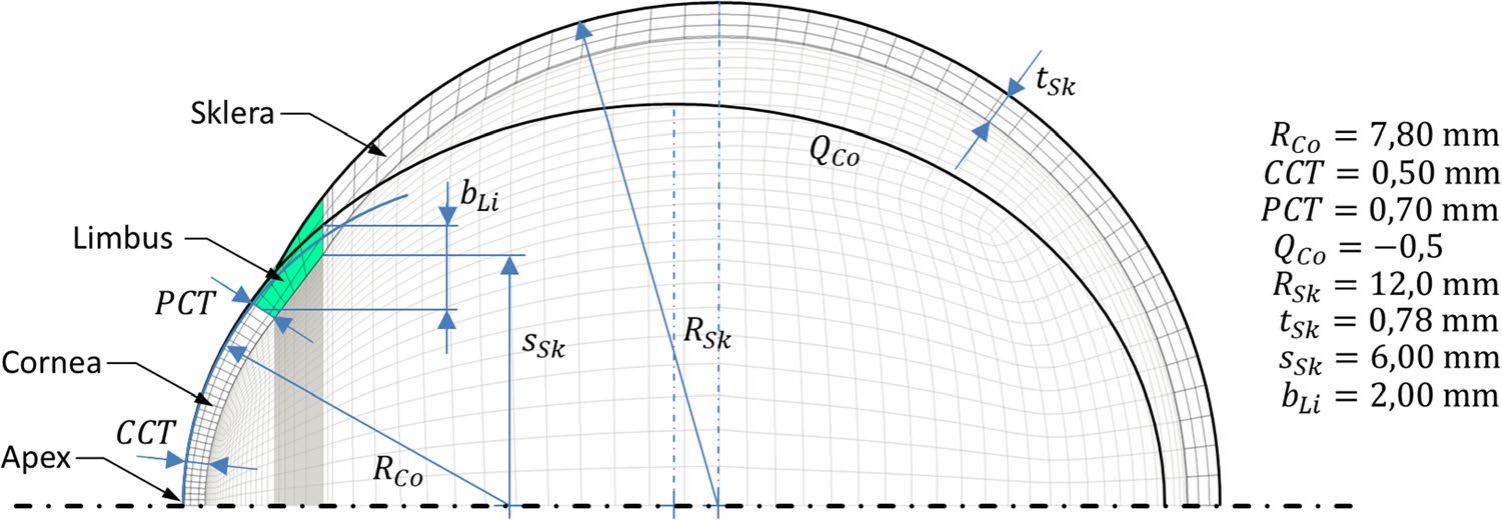
FE model of the eye. Lens and hydrostatic fluid elements are hidden for clarity. The geometric parameters are based on values according to (Woo et al. 1972a)

In order to enable a realistic computational simulation of non-contact tonometry, an accurate model of the human eye is necessary. The eye shell consists of two dominant primary geometries, see Fig. 1. In the proposed model, the cornea is represented as an ellipsoid. Its major semiaxis is coincident with the symmetry axis of the eye. The sclera is simplified to a sphere. Due to the symmetrical structure, the eye can be transferred into a quarter model. According to reality, the cornea has a lower thickness in the center than in the peripheral region. The limbus forms a 2 mm wide transition from the cornea to the sclera at about 4.6 mm. The thickness of the sclera is kept constant for simplification.

**Fig. 2:**
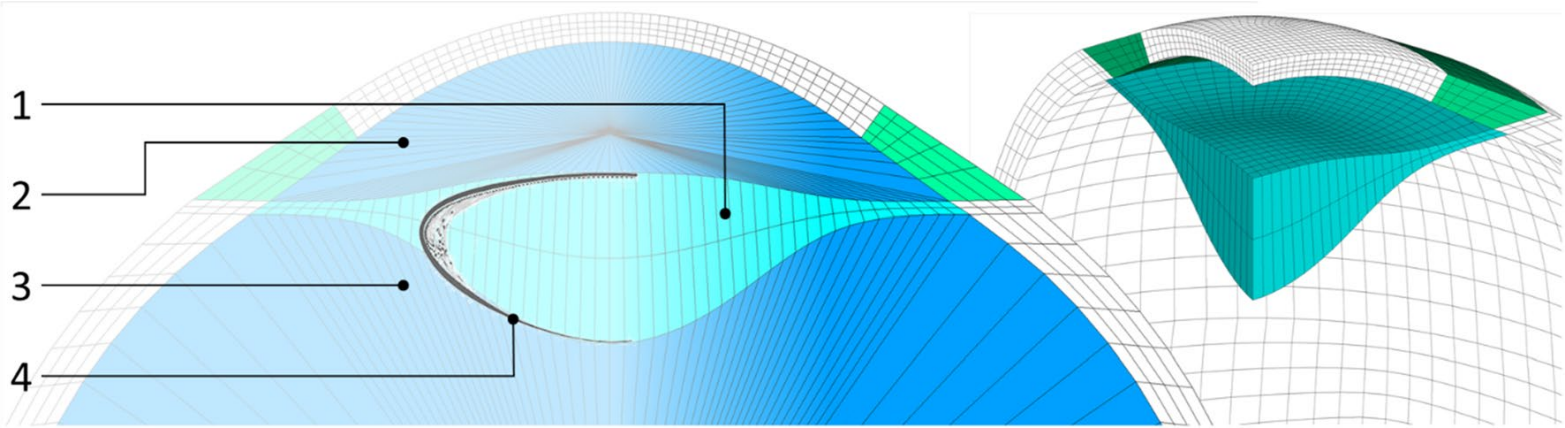
Comparison of the lens (1) in the FE model with the typical lens geometry (4) according to (Freyler 1985). The lens separates the interior of the eye into the anterior and posterior chamber volume (2) and the volume of the vitreous body (3)

For the process of NCT, the system response of the eye is important. This includes not only the deformation of the eye shell but also the varying IOP. The lens (1) separates the interior of the eye into two Sects. (2, 3), see Fig. 2. This separation leads to different intraocular pressures in the sections during the NCT. Accordingly, the modeling of the interior of the eye is also of high importance. The lens is rotationally symmetrical to the symmetry axis of the eye. Its front side is flattened compared to the back side. For simplicity, the lens, zonula fibers and trabecular meshwork are modeled as one volume (1). In the model, it is suspended underneath the limbus. The iris is neglected with respect to a very small mechanical influence on the corneal deformation. The anterior and posterior chambers of the eye are combined into one volume (2). This is meshed with hydrostatic fluid elements, which calculate an IOP increase depending on the displaced volume. The vitreous body (3) is also meshed with hydrostatic fluid elements.

Mapped mesh technique has been used to discretize the geometry. Hexahedral 8-node elements represent the solid components: cornea, limbus, sclera and lens. In order to reduce the influence of the discretization on the results, a mesh influence study is performed according to Celik et al. (2008). The target parameters are the corneal deflection amplitude $DefA$, the distance of the bending maxima $PD$ and the ${IOP}_{\mathrm{AC}}$ in the anterior chamber of the eye. They are determined at the time of the maximum pressure pulse applied to the cornea. For the applied mesh, grid convergence indices (GCI) of less than 1% are obtained for all three target variables using 41,184 solid elements and 10,140 fluid elements.

The used fluid elements are specifically designed and implemented in ANSYS® Mechanical APDL for modeling fluid-filled volumes without inlet or outlet. The elements have a pyramid shape, where the nodes of the bases are coincident with the nodes of the solid elements forming the boundary of the fluid volume and share their displacement degrees of freedom. The node at the top of the pyramid has pressure as the only degree of freedom and is shared by all elements of this type. Deformations of the limiting solid elements cause a change in the volume of the hydrostatic elements and thus a change in the pressure at the pressure node. The pressure determined in this way is constant for all elements and acts as a force per unit area on the base surface of the pyramid. The elements form a fluid volume without pressure or density gradients and without inertial forces. The compressibility of the fluid volume is set via the compression modulus (ANSYS, Inc 2015).

**Fig. 3:**
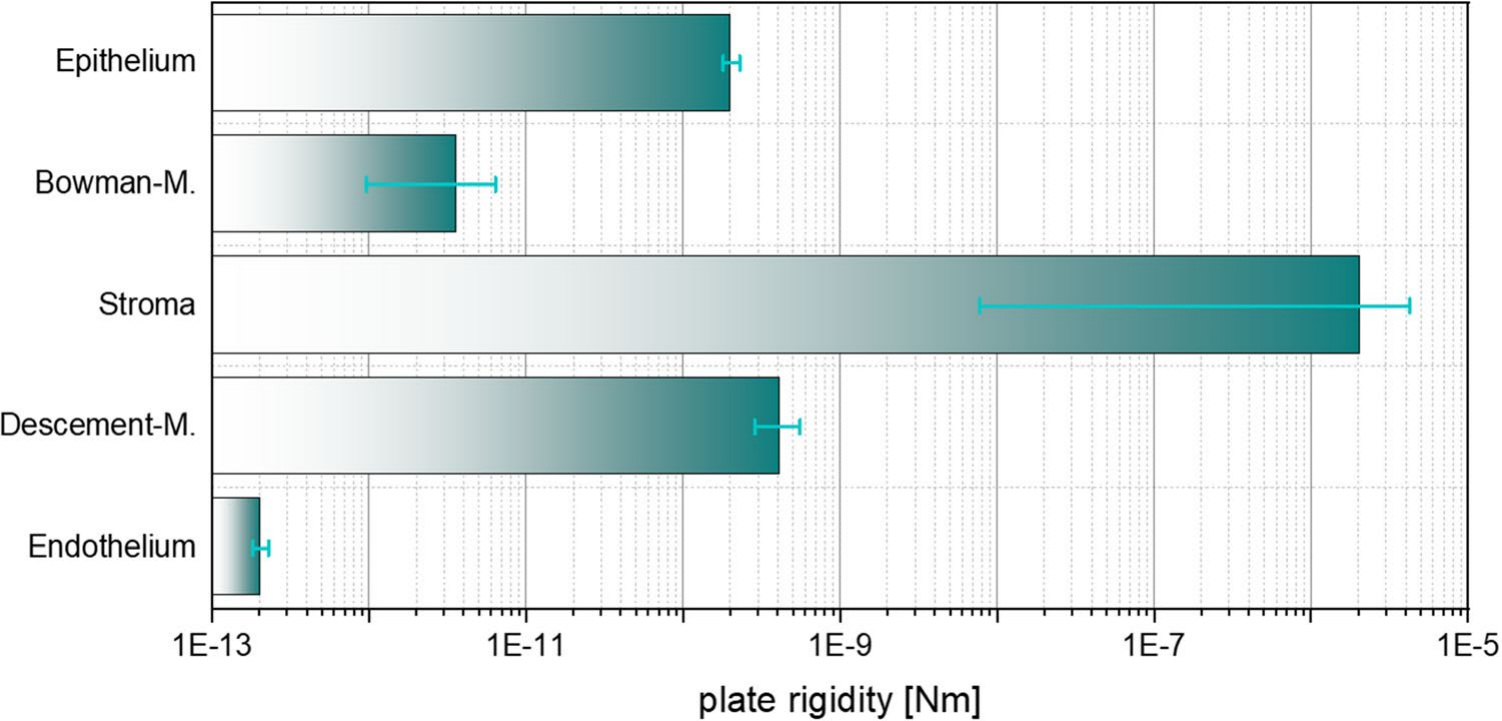
Plate rigidity of the individual layers of the cornea in logarithmic scaling. The error bars result from the ranges of variation of layer thickness and elastic modulus in the literature

To represent the complex material behavior of the cornea, consisting of extracellular matrix and collagen fibers, the model according to Sánchez, Moutsouris and Pandolfi (2014) was used in a slightly modified manner and then implemented into ANSYS® using the USERMAT subroutine. It is assumed that the stroma has the main mechanical influence during NCT and that the remaining thin layers are negligible. Instead of representing them, they are added to the stroma and receive the same properties, which is a common approach, but has never been fully substantiated to the best of the authors’ knowledge. At the microscopic level, the cornea can be divided into six layers. On the outside of the cornea is the epithelium, which consists of several cell layers. With a thickness of approx. 50 µm (Reinstein et al. 2008), it forms the outermost protective shell of the cornea, and according to McKee et al. (2011); Straehla et al. (2010), it has a modulus of elasticity of ${E}_{\mathrm{Epi}}=13-16.5\,\mathrm{kPa}$. Below the epithelium is the Bowman membrane measuring $8 - 15 \mu \mathrm{m}$ (Reinstein et al. 2008; Komai and Ushiki 1991; Kohlhaas et al. 2006), a dense tissue of interwoven collagen fibrils (Reinstein et al. 2008; Komai and Ushiki 1991; Meek and Knupp 2015) with a modulus of elasticity of ${E}_{\mathrm{Bow}}=17 \, \mathrm{kPa}$ as determined by Seiler et al. (1992). Mainly responsible for the mechanical behavior is the central stroma (Meek and Knupp 2015; White et al. 2017; Pinsky and Datye 1991; Pandolfi and Manganiello 2006; Winkler et al. 2011; Liu et al. 2014) which occupies almost 90% of the corneal thickness and has elastic moduli of ${E}_{\mathrm{Stroma}}=0.7-253 \, \mathrm{kPa}$ (Seiler et al. 1992; Winkler et al. 2011). On its inner side, the Dua membrane is attached, which has a thickness of about 10 µm and consists of densely packed and irregularly oriented collagen fibrils (Dua et al. 2013). The inner basement membrane is the Descemet's membrane, which is approximately 10 µm (Dua et al. 2013) thick and has elastic moduli of ${E}_{\text{Dua}}=2.57-5 \, \text{MPa}$ (Jue and Maurice 1986; Danielsen 2004). The endothelium demarcates the cornea from the anterior chamber of the eye with a thickness of approximately 5 µm (Tuft and Coster 1990) and elastic moduli similar to those of the epithelium. Using Eq. (1) for calculating the bending stiffness of a plate, the associated plate rigidity can be calculated from the above-mentioned layer thicknesses $t$ and elastic moduli $E$ according to Fig. 3. Comparing the logarithmically scaled bars, it is clear how mechanically significant the stroma is compared to the rest of the corneal layers, thus justifying the neglect of these layers in the mechanical analysis of the cornea's bending behavior.

1 $$K_{\mathrm{Pl}} = \frac{{E t^{3} }}{{12 \left( {1 - \nu^{2} } \right)}}$$

Here, we only recapitulate the major components of the material model proposed in Sánchez et al. (2014), which is mostly based on the assumption that there exists a hyperelastic strain energy density function of the structure

2 $$\psi = \psi_{\mathrm{vol}} \left( J \right) + \tilde{\psi }_{\mathrm{iso}} \left( {\tilde{I}_{1} } \right) + \mathop \sum \limits_{i = 1}^{n = 2} \tilde{\psi }_{\mathrm{ti}, i} \left( {\tilde{I}_{4,i}^{*} } \right)$$

wherein the volumetric part $\psi_{{{\text{vol}}}}$ is used here as penalty function to account for quasi-incompressibility. The energy density $\psi_{{{\text{iso}}}}$ describes the response of the isotropic matrix material, the function $\psi_{{{\text{ti}}}}$ the response of the collagen fibers. As deformation measures, the determinant $J$ of the deformation gradient ${\varvec{F}}$, the isochoric parts of the first principal invariant $\tilde{I}_{1}$ of the right Cauchy–Green tensor (in index notation for a fixated Cartesian coordinate system) $C_{jk} = F_{ij} F_{ik}$ and the isochoric part of the fourth modified invariant $\tilde{I}_{4}^{*}$ as a mixture of the fourth invariant $\tilde{I}_{4}$ defined in terms of $C_{jk}$ and the structural tensor $M_{jk} = a_{j} a_{k}$ with the fiber direction vector ${\varvec{a}}$ and the invariant $\tilde{I}_{1}$:

3 $$\tilde{I}_{4}^{*} = \kappa \tilde{I}_{1} + \left( {1 - 3\kappa } \right) \tilde{I}_{4}$$

with the position-dependent dispersion parameter $\kappa \in \left[ {0, 1/3} \right]$. The individual energy density functions are given by

4 $$\psi_{\mathrm{vol}} \left( J \right) = \frac{1}{4}K \left[ {J^{2} - 1 - 2 \ln \left( J \right)} \right]$$

5 $$\tilde{\psi}_{\mathrm{iso}} \left( {\tilde{I}_{1} } \right) = \frac{\mu }{2}\left( {\tilde{I}_{1} - 3} \right)$$

6 $$\tilde{\psi }_{\mathrm{ti}, i} \left( {\tilde{I}_{4,i}^{*} } \right) = - \frac{{k_{1} }}{{2k_{2} }} + \frac{{k_{1} }}{{2k_{2} }}\exp \left[ {k_{2} \left( {\tilde{I}_{4,i}^{*} - 1} \right)^{2} } \right] {\underbrace {{\left( 1 + K_{i}^{*} \sigma_{{\tilde{I}_{4,i}^{*} }}^{2} \right)}}_{a}}$$

wherein $a$ is defined as the second order term of the Taylor expansion of ${\stackrel{\sim }{\psi }}_{\mathrm{ti}, i}$ around ${\tilde{I }}_{4}^{*}$. Based on the energy density $\psi$, the Cauchy stress tensor entering the equilibrium equation as a basis of the structural boundary value problems considered later can be computed mainly as derivative of the energy with respect to the strains. The energy equations include the penalty parameter $K$, the components ${\mu }_{1}$ and ${\mu }_{2}$ of the shear modulus $\mu$ as well as the collagen fiber stiffness ${k}_{1}$ and the dimensionless material constant ${k}_{2}$ (Sánchez et al. 2014). The anisotropic part of the energy density function is calculated from the sum of the transversely isotropic fiber components for mainly two collagen fiber directions $n=2$. Represented are the two main directions, nasal–temporal and superior–inferior, with the location-dependent fiber dispersions corresponding to Simonini and Pandolfi (2015). Please note that the non-axisymmetric fiber orientation and dispersion are implemented directly in the material model through the position-dependent fiber direction vector ${\varvec{a}}$ and dispersion parameter $\kappa$. Since the difference between the first and second invariants ${\tilde{I }}_{1}$ and ${\tilde{I }}_{2}$ in the stretch range of the cornea during NCT is very small, this results in a low sensitivity with respect to the two associated parameters in the original model ${\mu }_{1}$ and ${\mu }_{2}$ and thus a potentially overrated sensitivity in the inverse problem. For this reason, we propose the simplification of the Mooney–Rivlin model, which was used in the original model (Sánchez et al. 2014) for the isotropic response, to the neo-Hookean model with only one parameter $\mu ={\mu }_{1}+{\mu }_{2}$ shown in Eq. (5) for the application to the eye. One further argument for the change to the neo-Hookean model becomes apparent when analyzing realistic parameters for ${\mu }_{2}$. Table 1 summarizes typical values from the literature for the parameters mentioned. They were inversely determined by the respective authors using different experimental data sets. As can be seen, in many cases negative values for ${\mu }_{2}$ are obtained rendering the isotropic energy function non-polyconvex and thus not automatically materially stable (Schröder et al. 2005). However, not satisfying such generalized convexity conditions may allow for an unphysical response and thus questionable numerical results. In the isotropic energy function, this problem is cured when the neo-Hookean model is applied where only positive values of $\mu$ are obtained resulting in a polyconvex isotropic energy density. Due to the fact that NCT, as a medical diagnostic tool, is designed to not induce damage to the tissue, we assume that the deformations during NCT are still in a physiological regime and thus not associated with microscopic damage and a resulting stress-softening response. Thus, the hyperelastic formulation (Sánchez et al. 2014) is considered. If supra-physiological deformation regimes are to be analyzed, convexified damage models (Balzani and Ortiz 2012) may be considered which have been also successfully applied for arterial walls with mainly two collagen reinforcements (Schmidt and Balzani 2016). But for NCT such loading regimes should in fact be avoided rendering a hyperelastic formulation a reasonable choice. Based on the parameter data in Table 1, we define our reference data set for healthy corneas (last line in Table 1). For the investigation of the accuracy of the inverse method described later, degraded properties are also applied. A pathological change of the cornea in keratoconus leads to the reduction of collagen fiber stiffness (Bron 1988). Accordingly, three degraded material data sets KK-I, KK-II and KK-III with a reduced fiber stiffness value ${k}_{1}$ are defined. Table 2 summarizes the material parameters used during virtual NCT. Here, we are interested in the analysis of a keratoconus-like disease in an early stage, and thus, it is included by weakening the material parameter value associated with the fiber stiffness. No additional adjustment of the initial geometry to the level of disease is considered which would appear at later stages of the disease.

**Table 1:** Inversely calculated material parameters from the literature and calculation of the resulting parameter ${\mu}={\mu}_{1}+{\mu}_{2}$. in—in vivo, ex—ex vivo, d—indentation, i—inflation, k—keratectomy, t—tensile test

Publication	$K \, [\mathrm{MPa}]$	${\mu }_{1} \, [\mathrm{MPa}]$	${\mu }_{2} \, [\mathrm{MPa}]$	$\mu \, [\mathrm{MPa}]$	${k}_{1} \, [\mathrm{MPa}]$	${k}_{2} [-]$	Exper. basis
Alastrué et al. (2006)	0.075	0.005	0	0.005	0.0049	103	Ex/i (Bryant and McDonnell 1996)
Pandolfi and Manganiello (2006)	5.500	1.055 7.555 0.00505 0.055	− 1.0455 7.550 − 0.005 − 0.005	0.0005 0.005 0.00005 0.050	0.250 0.175 0.055 0.055	50 15 16 14	Ex/t (Bryant et al. 1994) Ex/t (Hoeltzel et al. 1992) Ex/t (Zeng et al. 2001) Ex/t (Wollensak et al. 2003)
Petsche and Pinsky (2013)	–	0.00559	–	0.00559	0.638	314	in/d
Sánchez et al. (2014)	5.500	0.060 0.090	− 0.010 − 0.020	0.050 0.070	0.040	200	in/k
Reference values (healthy)	10	0.275	0	0.275	0.040	200

**Table 2:** Summary of the material parameters assigned to the simulation model

Component	Material model	Property
Cornea	Anisotropic, hyperelastic, incompressible	$K=10 \, \mathrm{MPa}$ $\mu =0.275 \, \mathrm{MPa}$ ${k}_{1}=0.04 \, \mathrm{MPa}$ (Healthy) ${k}_{1}=0.02 \, \mathrm{MPa}$ (KK-I) ${k}_{1}=0.01 \, \mathrm{MPa}$ (KK-II) ${k}_{1}=0.00 \, \mathrm{MPa}$ (KK-III) ${k}_{2}=200 \, \mathrm{MPa}$
Limbus	Isotropic, linear–elastic, incompressible	${E}_{\mathrm{Li}}=1.4 \, \mathrm{MPa}$, $\nu =0.49$
Sclera	Isotropic, linear–elastic, incompressible	${E}_{\mathrm{Sk}}=2.3 \, \mathrm{MPa}$, $\nu =0.49$
Lens	Isotropic, linear–elastic, incompressible	${E}_{\mathrm{Le}}=2.4 \, \mathrm{MPa}$, $\nu =0.49$
Aqueous humor	Incompressible	${K}_{\mathrm{W}}=2.0 \, \mathrm{GPa}$

Besides the cornea, the eye model contains further components, which receive reasonably simplified properties, namely linear–elastic, isotropic and incompressible material properties. From the investigations on scleral specimens by means of tensile tests by Friberg and Lace (1988), the inflation tests by Woo, Kobayashi, Schlegel and Lawrence (1972b) and Battaglioli and Kamm (1984) as well as from applanation analyses in Kobayashi et al. (1971), an averaged elastic modulus of the sclera of ${E}_{\mathrm{Sc}}=2.3 \, \mathrm{MPa}$ is obtained. As a link between cornea and sclera, the limbus obtains an averaged property of both components: ${E}_{\mathrm{Li}}=0.5\left({E}_{\mathrm{Co}}+{E}_{\mathrm{Sc}}\right)$. Here, corneal stiffness refers to the publication (Woo et al. 1972a) about the behavior of the eye shell under IOP. Lens capsule, cortex and nucleus as well as zonular fibers and trabecular meshwork are combined into one body. Its properties correspond to those from the studies of Danielsen (2004) on lens capsules. For the aqueous humor, the compression modulus of water is given by ${K}_{\mathrm{W}}=2.0 \, \mathrm{GPa}$, which is used by the hydrostatic fluid elements to calculate the resulting intraocular pressure evoked by the change of the fluid volume.

The specified boundary conditions are subdivided into the mounting of the eye, the intraocular pressure IOP and the pressure and shear stresses applied to the corneal surface by the NCT. The displacement and rotation-free positioning of the eye take place on its posterior surface. All nodes of the scleral outer surface that are within an angle of 30° to the symmetry axis of the eye geometry are fixed, i.e., the displacements are set to zero there. The symmetry-related simplification to a quarter model results in intersecting surfaces. These are provided with floating bearings in the direction perpendicular to the symmetry planes, according to the symmetry constraints. The fluid elements for representing the anterior chamber of the eye and the vitreous body receive the IOP as a volume force, prescribed in the elements in the form of a hydrostatic pressure. This hydrostatic pressure is fixed to $17.5 \, \mathrm{mmHg}$ during the determination of the stress-free geometry. Once the geometry is determined and the preload is applied, the transfer of the boundary condition IOP to a degree of freedom is done. This allows the IOP to increase during corneal deformation, as it does in reality. The pressure and shear stresses on the corneal surface caused by the NCT are applied in a location-, time- and deformation-dependent manner. For this purpose, analytic equations are used, which were determined on the basis of experimental and numerical flow investigations and have already been published in Muench et al. (2019). For the sake of completeness, we have attached the used equations Eqs. (15) and (16) as well as the coefficients to be used in Appendix A. To represent an almost steady development of the load profile, a stepwise simulation of the NCT is performed, i.e., in each load step the changing, non-axisymmetric load profile according to the analytic equation in Appendix A (cf. (Muench et al. 2019)) is applied.

As previously mentioned, the real eye is prestressed during tomographic or topographic in vivo measurements due to the IOP. The stress-free geometry is unknown. An FE model built from these data would have the geometry of the prestressed eye in the stress-free state. When the IOP is applied, the model deforms. This results in a deformed geometry. In order to reduce influences due to these deformations to a minimum, an iterative calculation of the stress-free geometry is performed according to the publication by Pandolfi and Holzapfel (2008). The distance of the nodes between the target geometry and the nodes under prestress is used as a criterion for termination. The criterion is fulfilled if this distance is less than or equal to $2 \mathrm{\mu m}$ for all nodes.

### Inverse method for linear parameters

To give a short recapitulation of the basic mathematical model, we would like to summarize the principle of the minimum of the total potential energy (TPE) and its finite element discretization considered here. Neglecting inertia, the total potential energy $\mathit\Pi$ of an elastic body in the reference configuration ${\mathcal{B}} \in {\text{R}}^{3}$, parameterized in terms of position vectors ${\varvec{X}}$**,** can be written as

7 $$\mathit{\Pi} \left( {\varvec{u}} \right) = \underbrace {{\int\limits_{{\mathcal{B}}} {\psi \left( {{\varvec{C}}({\varvec{F}}({\mathrm{Grad}}\,{\varvec{u}}} \right)))\,{\mathrm{dv}}} }}_{{ \mathrm{=:} \: \mathit{\Pi}_{\mathrm{int}} }}\underbrace {{ - \int\limits_{{\mathcal{B}}} {{\varvec{u}} \cdot \overline{\user2{b}}\,{\mathrm{dv}} - \mathop \smallint \limits_{{\partial {\mathcal{B}}_\fancyscript{t} }} {\varvec{u}} \cdot \overline{\user2{t}}\,{\mathrm{da}}} }}_{{ =: - \mathit{\Pi}_{\mathrm{ext}} }}$$

with ${\text{Grad}}\,{\varvec{u}} = \partial {\varvec{u}}/\partial {\varvec{X}}$ describing the derivative of the displacement field ${\varvec{u}}$ with respect to the position vector**,** the strain energy density $\psi$, the volume forces $\overline{\user2{b}}\,$ and the surface loads $\overline{\user2{t}}\,$. The variational principle states that the displacements in an elastic body adjust such that the total potential energy becomes minimal provided that Dirichlet and Neumann boundary conditions, $\varvec{u} = \varvec{\bar{u}}~{\text{on}}~\partial {\mathcal{B}}_{\mathrm{u}}$ and $\varvec{t} = \varvec{\bar{t}}~{\text{on}}~\partial {\mathcal{B}}_{\mathrm{t}}$, respectively, are fulfilled. The necessary condition is that the variation of the potential energy vanishes, i.e.,

8 $$ \delta \mathit\Pi = \int\limits_{{\mathcal{B}}} {\varvec{P} \cdot {\mathrm{Grad}}\,\delta \varvec{u} \, \mathrm{dV}} - \int\limits_{{\mathcal{B}}} {\delta \varvec{u} \cdot \varvec{\bar{b}}\, \mathrm{dV}} - \int\limits_{{\partial {\mathcal{B}}_\fancyscript{t} }} {\delta \varvec{u} \cdot \varvec{\bar{t}}\, \mathrm{dA} = \mathrm{0},}$$

for arbitrary variations ${\updelta }{\varvec{u}}$ satisfying homogeneous boundary conditions on $\partial {\mathcal{B}}_{\mathrm{u}}$. Herein, the first Piola–Kirchhoff stress tensor reads ${\varvec{P}}=\frac{\delta \psi }{\delta F}$, which can be reformulated in terms of the Cauchy stress tensor by ${\varvec{P}}=J{\varvec{\sigma}}{{\varvec{F}}}^{-\mathrm{T}}$. Thus, the first additive term in (8) modifies to ${\int }_{\mathcal{B}}{\varvec{P}}\cdot \mathrm{Grad\,\delta }{\varvec{u}}\hspace{0.17em}\mathrm{dV}= {\int }_{{V}_{e}}\mathrm{Grad\,\delta }{\varvec{u}}\hspace{0.17em}{{\varvec{F}}}^{-1}\cdot{\varvec{\sigma}}\boldsymbol{ }\,\mathrm{dv}$ with ${V}_{e}$ denoting the element volume in the current configuration. Now, a classical finite element ansatz is introduced for the displacements ${{\varvec{u}}}^{\mathrm{h}}={\varvec{N}}{\varvec{d}}$ in terms of the nodal displacements ${\varvec{d}}$ and the classical finite element matrix ***N*** including the shape functions, and accordingly for the variations of the displacements $\updelta {{\varvec{u}}}^{\mathrm{h}}={\varvec{N}}\updelta {\varvec{d}}$. Using Voigt notation, this results in the finite element representation of Eq. (8) as

9 $$\delta \mathit{\Pi} ^{\mathrm{h}} \: \mathrm{=} \: \sum\limits_{e} {\delta \varvec{d}^{{e\mathrm{T}}} \left( {\int\limits_{{V_{e} }} {\varvec{B}^{{e\mathrm{T}}} ~\varvec{\sigma }\,\mathrm{dv}} - \int\limits_{{{\mathcal{B}}_{e} }} {\varvec{N}^{{e\mathrm{T}}} \varvec{\bar{b}}\,\mathrm{dV}} - \int\limits_{{\partial {\mathcal{B}}_{{e\fancyscript{t}}} }} {\varvec{N}^{{e\mathrm{T}}} \varvec{\bar{t}}\,\mathrm{dA}} } \right) \mathrm{=} \: \mathrm{0},}$$

wherein $\updelta {{\varvec{d}}}^{e}$, ${{\varvec{N}}}^{e}$ and ${{\varvec{B}}}^{e}$ correspond to the element-wise matrices. Representing the summation in terms of global matrix multiplications, i.e., introduction of a global vector of displacement variations $\updelta \widehat{{\varvec{u}}}$, which contains all nodal variations, and making use of the fact that the variations shall be arbitrary, enables the reformulation of (9) as a discrete residual matrix defined as difference between discrete internal ${{\varvec{f}}}^{\mathrm{int}}\left(\widehat{{\varvec{u}}}, \boldsymbol{\alpha }\right)$ and external ${{\varvec{f}}}^{\mathrm{ext}}$ force matrices. By iteratively changing the nodal displacement matrix $\widehat{{\varvec{u}}}$ until the residual matrix approaches zero, mechanical equilibrium can be achieved. The discrete residual ${\varvec{R}}$ is calculated as

10 $${\varvec{R}} = {\varvec{f}}^{{{\text{int}}}} \left( {\widehat{\user2{u}}, {\varvec{\alpha}}} \right) - {\varvec{f}}^{{{\text{ext}}}}$$

with the material parameter matrix $\boldsymbol{\alpha }={\left(K, \mu , {k}_{1}, {k}_{2}\right)}^{\mathrm{T}}$. The matrix with discrete nodal entries of internal forces is obtained by standard assembly procedure of the element-wise column matrix

11 $$\varvec{f}_{e}^{{\text{int} }} \left( {\varvec{\widehat{u}},~\varvec{\alpha }} \right) = \int\limits_{{V_{e} }} {\varvec{B}^{{e\mathrm{T}}} ~\varvec{\sigma }\left( {\varvec{\widehat{u}},~\varvec{\alpha }} \right)~\mathrm{dv}} = \mathop \sum \limits_{{i = 1}}^{3} \alpha _{i} \underbrace {{\int\limits_{{V_{e} }} {\varvec{B}^{{e\mathrm{T}}} ~\varvec{\widehat{\sigma }}_{i} \left( {\varvec{\widehat{u}}} \right)~\mathrm{dv}} }}_{{c_{i} }}$$

which is identified as integral over the element volume ${V}_{e}$ of the standard ${{\varvec{B}}}^{e}$**-**matrix multiplied by the Cauchy stress matrix ${\varvec{\sigma}}$ in Voigt notation. The ${{\varvec{B}}}^{e}$**-**matrix is an element-specific matrix and contains the derivatives of the element ansatz functions (Zienkiewicz and Taylor 2005). The stresses ${\varvec{\sigma}}$ are computed as matrix representation of the tensor product ${{\varvec{\sigma}}}_{\mathrm{jk}}=2/{J}\: {F}_{\mathrm{jm}} \: \partial \mathit{\Psi} /\partial {C}_{\mathrm{mp}} \: {F}_{\mathrm{pk}}^{\mathrm{T}}$. In the model proposed by Sánchez, Moutsouris and Pandolfi (2014), the parameters ${K},\:\mu$ and ${k}_{1}$ enter the strain energy density linearly in three separate additive terms, and thus, the internal forces can be split into three additive terms ${\alpha}_{i}{c}_{i}$ with ${i}=1,2,3$ and ${\alpha}_{1}={K}, \: {\alpha}_{2}=\mu, \: {\alpha}_{3}={k}_{1}$. Herein, ${{\varvec{c}}}_{{i}}$ represents the column matrix as defined in Eq. (11). Thereby, the residuum turns out to be a linear function in these three parameters which is why we refer to these as *linear parameters*. In order to compute these from solving the inverse problem, the nodal displacement matrix $\widehat{{\varvec{u}}}$ is assumed to be fully known here from the NCT measurement. Note that currently the full-field data cannot yet be measured due to technical limitations in the NCT devices. Therefore, we propose to construct an approach which provides a reasonable approximation of the full-field kinematics, but this will be addressed later in this paper. Provided that the displacements as well as the external forces are known, the material parameters $\boldsymbol{\alpha }$ remain the only unknowns in the residuum ${\varvec{R}}$. Assuming that the *nonlinear parameter* ${k}_{2}$, which appears nonlinearly in the residuum, is known during the identification of the linear parameters, the residuum can be rewritten as

12 $${\varvec{R}} = K{\varvec{c}}_{{\text{K}}} + \mu {\varvec{c}}_{{\upmu }} + k_{1} {\varvec{c}}_{{{\text{k}}_{1} }} - {\varvec{f}}^{{{\text{ext}}}}$$

Herein, the matrix ${{\varvec{c}}}_{i}$, i.e., ${\varvec{c}}_{{\text{K}}} {: = }{\varvec{c}}_{1} ,{\varvec{c}}_{{\upmu }} {: = }{\varvec{c}}_{2} ,{\varvec{c}}_{{{\text{k}}_{1} }} {: = }{\varvec{c}}_{3}$, contains the result of the integral evaluation and is constant for a given displacement field. The basic idea of the equilibrium gap method (EGM) is that the parameters can be identified by minimizing ${\varvec{R}}$, with the main advantage that the residuum has only to be evaluated. This is in contrast to updating procedures where the expensive forward problem needs to be solved within each optimization step. The main interpretation of the EGM is that the obtained optimal choice of the parameters is associated with the best possible achievement of equilibrium given a specific displacement field, external forces and material model. Since neither the material model nor the measurement of displacements or external forces can never be precise, exact equilibrium will never be reached. However, in a post-processing step, the forward problem may be solved using the identified parameters, and then, the calculated displacement field can be compared with the measured one. Then, if the similarity is found insufficient, improvements can only be achieved by improving the material model as the EGM already resulted in the optimal values of parameters. In order to exploit the fact that the residuum is linear in the linear parameters, minimization of ${{\varvec{R}}}^{\mathrm{T}}{\varvec{R}}$ renders a quadratic optimization problem still in line with the original idea of the EGM. The main advantage is that this quadratic and thus convex problem has a unique solution and enables thereby a unique identification of at least the linear parameters. The property that if the derivative of the strain energy function with respect to the deformation tensor is linearly dependent on the material parameters, these can be determined uniquely and directly, has already been observed by Cottin, Felgenhauer and Natke (1984). Due to the fact that the optimization problem is convex, the necessary and sufficient conditions for the optimum are

13 $$\frac{{\delta {\varvec{R}}^{{\text{T}}} {\varvec{R}}}}{\delta K} = 0,\frac{{\delta {\varvec{R}}^{{\text{T}}} {\varvec{R}}}}{\delta \mu } = 0\;{\text{and}}\;\frac{{\delta {\varvec{R}}^{{\text{T}}} {\varvec{R}}}}{{\delta k_{1} }} = 0$$

Since ${{\varvec{R}}}^{\mathrm{T}}{\varvec{R}}$ is quadratic, the derivatives in Eq. (13) will be linear in the parameters $K, \mu , {k}_{1}$, and thus, only a linear system of three equations has to be solved to compute the optimal values of linear parameters. A direct consequence is that in principle no iterative numerical optimization procedure is necessary rendering the approach real-time capable. However, usually constraints regarding the parameters need to be considered, for instance, here $K\ge 0, \mu \ge 0, {k}_{1}\ge 0$ due to convexity conditions, and thus, it may happen that the conditions in Eq. (13) would be fulfilled within the precluded parameter regions. Then, an iterative numerical procedure is required to track the parameter space boundaries until the optimum of ${{\varvec{R}}}^{\mathrm{T}}{\varvec{R}}$ is found. But this case did not appear in our analysis, even if the fact that the calculation of the objective function does not include the expensive numerical solution of boundary value problems still renders the numerical procedure almost real-time capable. Note that the implementation using standard finite element software for the calculation of the residuum can be obtained in a straightforward, noninvasive manner. Then, for the calculation of the individual matrices ${{\varvec{c}}}_{i}$, simply the associated parameter is set to ${\alpha }_{i}=1$ and the remaining parameters are set to ${\alpha }_{j}=0, j\ne i$. Furthermore, the external forces are set to zero. Then, the FE software is called and the returned discrete residuum matrix represents the matrix ${{\varvec{c}}}_{i}$. For example, if the matrix associated with $K$ has to be computed, the FE software can directly provide ${{\varvec{c}}}_{\mathrm{K}}={\varvec{R}}\boldsymbol{ }\left(K=1, \mu =0, {k}_{1}=0,{{\varvec{f}}}^{\mathrm{ext}}=\varvec{0}\right)$.

### Inverse method for the nonlinear parameter

The dimensionless material parameter ${k}_{2}$, which was previously considered known, has a nonlinear effect on the transversely isotropic part of the strain energy function in Eq. (6) and thus on the optimization problem as part of the parameter identification. Accordingly, an unambiguous determination according to the presented approach is not possible. The parameter mainly influences the nonlinearity of the stress–strain curve of the collagen fibers. Consequently, several supporting points of different deformation states are necessary for its determination. In Perotti et al. (2017), it is proposed to determine such parameters by an additional identification loop. For an initialized and fixed ${k}_{2}$, the determination of the linear material parameters ${\alpha }_{i}$ is separately performed for $m\ge 3$ different deformation states. Thereby, a set of separate values of linear parameters for each deformation state is obtained. Of course, eventually, a fixated set of parameter values should be obtained no matter which deformation state. Therefore, the similarity between the separate material parameter sets serves as a quality criterion for the selected ${k}_{2}$ (Perotti et al. 2017). To define a suitable criterion, we propose the sum of the standard deviations ${\sigma }_{\mathrm{Dev}}$ of the material parameter sets related to their mean values $\langle \cdot \rangle$, and thus, an external objective function is defined as

14 $$f_{\mathrm{rel}} \left( {k_{2} } \right) = \mathop \sum \limits_{i = 1}^{3} \frac{{\sigma_{{\mathrm{Dev}, \alpha_{i} }} \left( {k_{2} } \right)}}{{ \alpha_{i} \left( {k_{2} } \right)}} .$$

This function depends on ${k}_{2}$ since the identification of the separate linear parameter sets depends on the specific choice of ${k}_{2}$. By minimizing ${f}_{\mathrm{rel}}$ with respect to ${k}_{2}$, the specific value for the nonlinear parameter is obtained which enables an optimal representation of the degree of nonlinearity of the material response. Note that this overall procedure represents a nested optimization problem because for each evaluation of ${f}_{\mathrm{rel}}$ several optimization problems for the linear parameters according to the different deformation states need to be solved. Since ${f}_{\mathrm{rel}}$ is usually not convex in ${k}_{2}$, neither a unique identification nor the calculation of the global minimum can be guaranteed. In addition to that, a global non-convex optimizer needs to be applied rendering the external minimization problem comparatively expensive. However, the identification of ${k}_{2}$ does not necessarily need to be performed patient-specific. On the contrary, it is rather reasonable to identify this nonlinear parameter first based on experimental data, and then, while keeping ${k}_{2}$ fixated as part of the model formulation, only the linear parameters are identified patient-specifically in real time.

### Morphing approach for the construction of representative 3D full-field kinematics

**Fig. 4:**
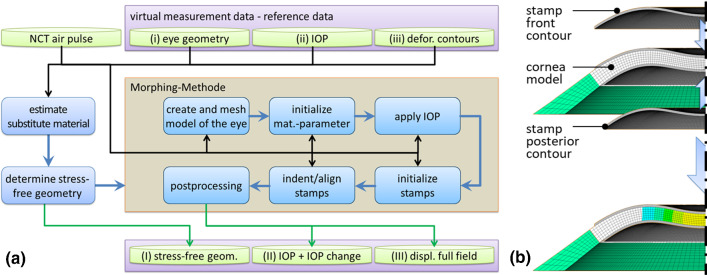
**a** Process diagram of the transformation of measurement data (geometry, IOP, deformation contours) into the input data required for the inverse method (stress-free geometry, IOP including the IOP change, full-field displacement) using the mechanical morphing approach and **b** illustration of the principle of the mechanical morphing approach. Virtual stamps force the corneal model to deform

The previous approach is based on the knowledge of the nodal displacement matrix $\widehat{{\varvec{u}}}$. Since no measurement system is available so far to measure the full field during air pulse tonometry, the displacement matrix must be approximated otherwise. We propose the following mechanical morphing approach to make use of the time-resolved deformation contours of the anterior and posterior corneal surface determined with the Corvis® ST in such a way that a representative full field is obtained which is sufficiently comparable to the real full-field displacement to be able to determine material parameters according to the inverse approach described above. The (i) basic geometry of the eye, the (ii) IOP and the (iii) deformation contours are assumed to be given by the Corvis® ST and enter the approach as input data. From the geometry data, an eye model similar to the one previously explained is created and meshed. Under the assumption that the deformation behavior is essentially characterized by the incompressibility condition and since at this point no information is available regarding the mechanical properties of the cornea belonging to the data, the material is assumed with linear–elastic properties. According to Pandolfi and Holzapfel (2008), the stress-free geometry is determined from the model under IOP. Then the 2D deformation contours are imported and rotationally extracted to 3D stamps. The stamp of the front deformation contour is indented according to the deflection amplitude $DefA$. Then the stamp of the posterior deformation contour is aligned with the anterior stamp according to the central corneal thickness CCT. The stamps and the cornea are both provided with contact elements on the surface. These enable vertical force transmission so that the new corneal shape is imprinted. At the same time, the cornea can move horizontally by sliding. This is important because the Corvis® ST data only provides the information of the contour, not the displacements of specific points. Thereby, the eye model contour is transferred to the measured contour which is why we refer to this process as mechanical morphing approach. During the process, all model nodes take a displacement assumed to be similar to reality. These can then be read out as a representative displacement matrix ${\widehat{{\varvec{u}}}}_{\mathrm{morph}}$. The process is shown in Fig. 4a and illustrated in Fig. 4b. Note that the incompressibility of the cornea can be enforced in the morphing process in a straightforward manner by using classical approaches like the penalty method. Therefore, sensitivities in the material model resulting from compressible deformations can a priori be excluded.

In addition, the deformation of the cornea leads to a pressure increase in the fluid-filled interior of the eye. This IOP change is a non-negligible component of the external forces acting on the cornea and is relevant for the inverse parameter identification. Thus, the mechanical morphing approach has transformed the input variables (i) eye geometry, (ii) IOP and (iii) deformation contours into the output variables (I) stress-free geometry, (II) IOP including the IOP change and (III) the approximated full-field displacement ${\widehat{{\varvec{u}}}}_{\mathrm{morph}}$.

In order to be able to analyze the performance of this approach, the reference displacement matrices already obtained from the NCT simulation are used to construct the according 2D deformation contours as reference, because these would be obtained from the NCT measurement. For each reference data set, one contour for the front and back surface is created, and then, only these data are used as virtual experimental data instead of the full-field kinematics.

## Results

**Fig. 5:**
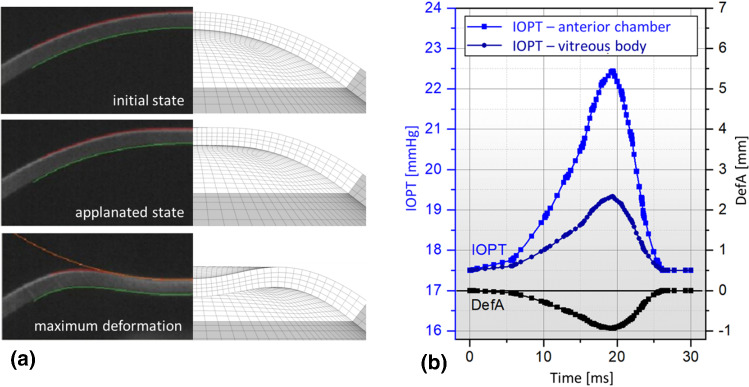
**a** Comparison of the simulated deformation behavior with the deformation states of the cornea recorded by Corvis® ST in sectional view according (Long et al. 2015) and **b** the calculated profile of the intraocular pressures in the anterior chamber and vitreous body as a function of the deflection amplitude ${DefA}$

The eye model is now used for the virtual NCT examination. Similar to the real eye, the model reacts with a corneal deformation to the applied air pulse. Figure 5a compares the typical deformation behavior of a cornea during NCT with the simulation results. The images of the real cornea are based on Corvis® ST measurements on healthy patients, performed in Long et al. (2015). The comparison shows a high qualitative similarity. This similarity indicates that the behavior of our FE approach, and thus the extracted representative displacements, can be considered realistic and thus representative. Furthermore, the indentation of the cornea leads to an increase in IOP due to the reduction of the anterior chamber volume. This reaction is shown in Fig. 5b. Note the decoupling of the anterior chamber from the vitreous body caused by the lens. Since the lens surface is smaller on the anterior chamber side than on the posterior side, the pressure in the anterior chamber increases much more. This increase counteracts corneal deformation, which would be greater in the absence of IOP response. It follows that consideration of the lens is highly important for NCT simulation. The results of the typical Corvis® ST parameters $DefA$ and $PD$ as well as the eye pressures in the anterior chamber ${IOP}_{\mathrm{AC}}$ and the vitreous body ${IOP}_{\mathrm{VB}}$ are summarized for the time point of maximum indentation in Table 3.

**Table 3:** Results calculated with the FE model at the time of maximum indentation depth for the 4 predefined material sets

Material set	$DefA \, [\mathrm{mm}]$	$PD \, [\mathrm{mm}]$	${IOP}_{\mathrm{AC}} \, [\mathrm{mmHg}]$	${IOP}_{\mathrm{VB}} \, [\mathrm{mmHg}]$
Healthy	$0.944$	4.870 ± 0.108	23.264	19.564
KK-I	$1.019$	5.068 ± 0.108	23.792	19.781
KK-II	$1.058$	5.061 ± 0.108	23.989	19.864
KK-III	$1.193$	5.047 ± 0.108	24.412	20.052

The basis for the verification of the inverse method is the synthetic measurement data, which were extracted from the NCT simulation. In addition to the stress-free geometry, three deformation states are used. State 1 is the applanation state, state 2 defines the deformation state in the middle between applanation and maximum deformation, and state 3 describes the maximum deformation. For these three states, the full-field displacement for each material data set (H, KK-I, KK-II, KK-III) is extracted. This results in 12 reference data sets which can be used to measure the performance of the method.

### Test with linear parameters

The solution of the system of Eq. (13) with directly inserting the full-field displacement data and the externally acting forces provides the resulting parameters summarized in Table 4. Since this investigation is intended to provide the proof of function for the linear parameters, the nonlinear parameter ${k}_{2}$ was kept constant according to the reference value of 200, which was identified in the following subsection. The inverse approach is shown to work very accurately based on the reference data for all three deformation conditions. The identification time was ${t}_{\mathrm{EGM}}\approx 20 \,\mathrm{s}$ using 1 core running at 3,2 GHz (Intel i7-8700). In comparison, a single forward solve of the NCT investigation takes ${t}_{\mathrm{FEM}} >6 \,\mathrm{h}$ using 1 core at 3,2 GHz (Intel i7-8700) without previous calculation of stress-free geometry and ${t}_{\mathrm{FEM}} >11 \,\mathrm{h}$ with calculation of the stress-free geometry, respectively. This underlines the advantage of incorporating a non-updating scheme for the inverse problem.

**Table 4:**
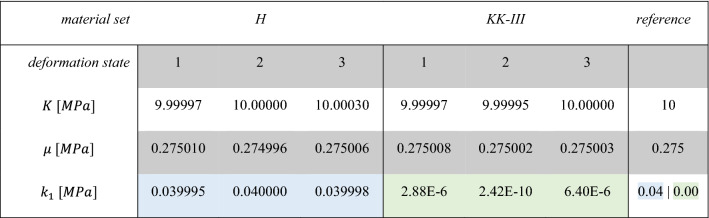
Comparison of the linear material parameters identified inversely for different deformation states to the reference values for a constant ${{k}}_{2}=200$

### Test with the nonlinear parameter

**Fig. 6:**
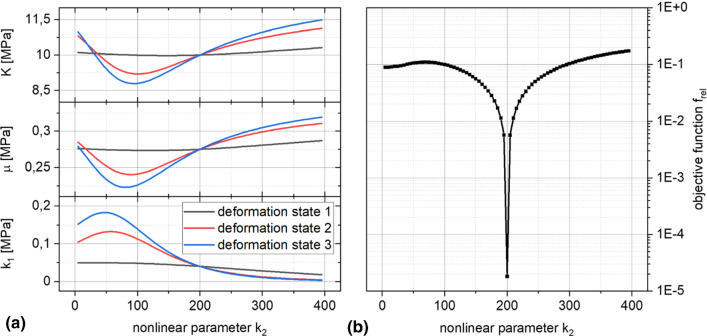
**a** Evolution of the linear material parameters as a function of the nonlinear parameter ${k}_{2}$ and **b** evolution of the error criterion ${f}_{\mathrm{rel}}$ according to Eq. (14) over ${k}_{2}$

**Fig. 7:**
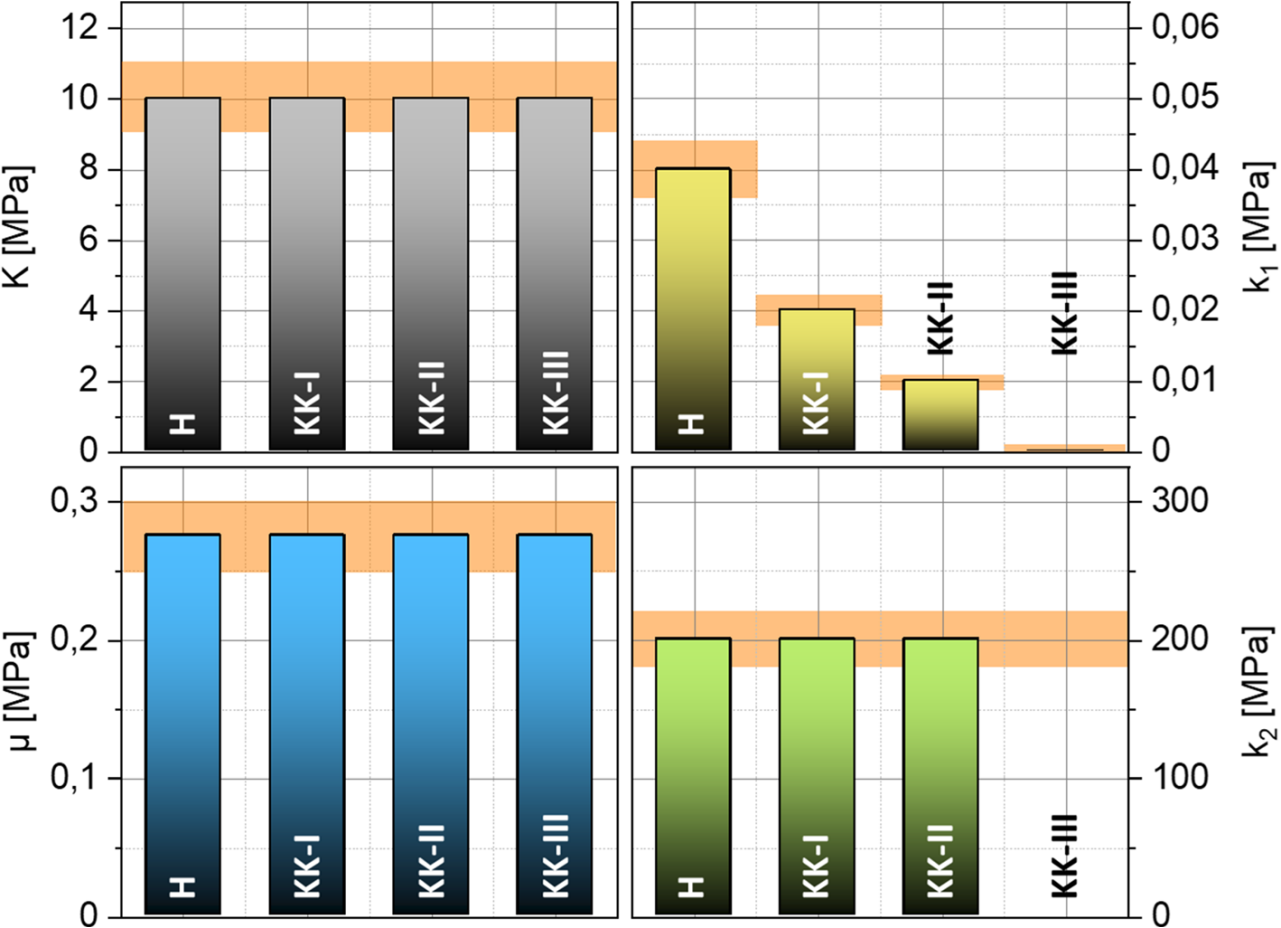
Inversely identified material parameters of the 4 reference data sets. The diagram shows the ± 10% deviation band from the reference value (orange)

To identify the nonlinear parameter ${k}_{2}$, the linear parameters are determined as a function of the varied parameter ${k}_{2}$. The results for the healthy reference data set are shown in Fig. 6a for the linear parameters $K$, $\mu$ and ${k}_{1}$ for the three deformation states. It also shows the nonlinear influence of ${k}_{2}$. The curves of the three states intersect at ${k}_{2} = 200$, where apparently the deviations of the deformation-state-dependent linear parameter sets are minimal. Figure 6b shows the behavior of the objective function according to Eq. (14). It shows the intersection point recognizable in Fig. 6a as a global minimum for ${k}_{2} = 200$, as well as a local minimum at $k_{2} \to 0$. Quantitatively the global minimum differs clearly from the local one, nevertheless a grid search from near zero upwards seems to be recommended for the secure identification of ${k}_{2}$. The linear parameters associated with the identified, optimal value for ${k}_{2}$ are presented in Fig. 7. The entire grid search in the range $10\le {k}_{2}\le 400$ with step size 10 took ${t}_{\mathrm{EGM},\mathrm{ NL}} \approx 720 \,\mathrm{s}$ when running the EGM in parallel on 3 cores at 3,2 GHz (Intel i7-8700). The comparable forward simulation would have required ${t}_{\mathrm{FEM}}\approx 10 \,\mathrm{d}$.

Figure 6a presents the results of the parameter identification for all 4 reference data sets H, K-I, K-II and K-III. Similar to the identification of the linear parameters, the results of the identification with nonlinear parameter *k*_2_ are again quite accurate. As before, the fiber stiffness parameter ${k}_{1}$ follows the predefined degradation. The new nonlinear and dimensionless material parameter ${k}_{2}$, which is now included in the identification, is correctly determined for the material parameter sets H, K-I and K-II. For the reference data set K-III, no identification is achieved with reference to the ${k}_{1}\approx 0$. Equation (6) shows that ${k}_{1}\approx 0$ switches off the transversely isotropic part of the strain energy density function, in which ${k}_{2}$ is also located. Thus, ${k}_{2}$ does not influence the minimization of the residuals anymore.

### Influence of noise

**Fig. 8:**
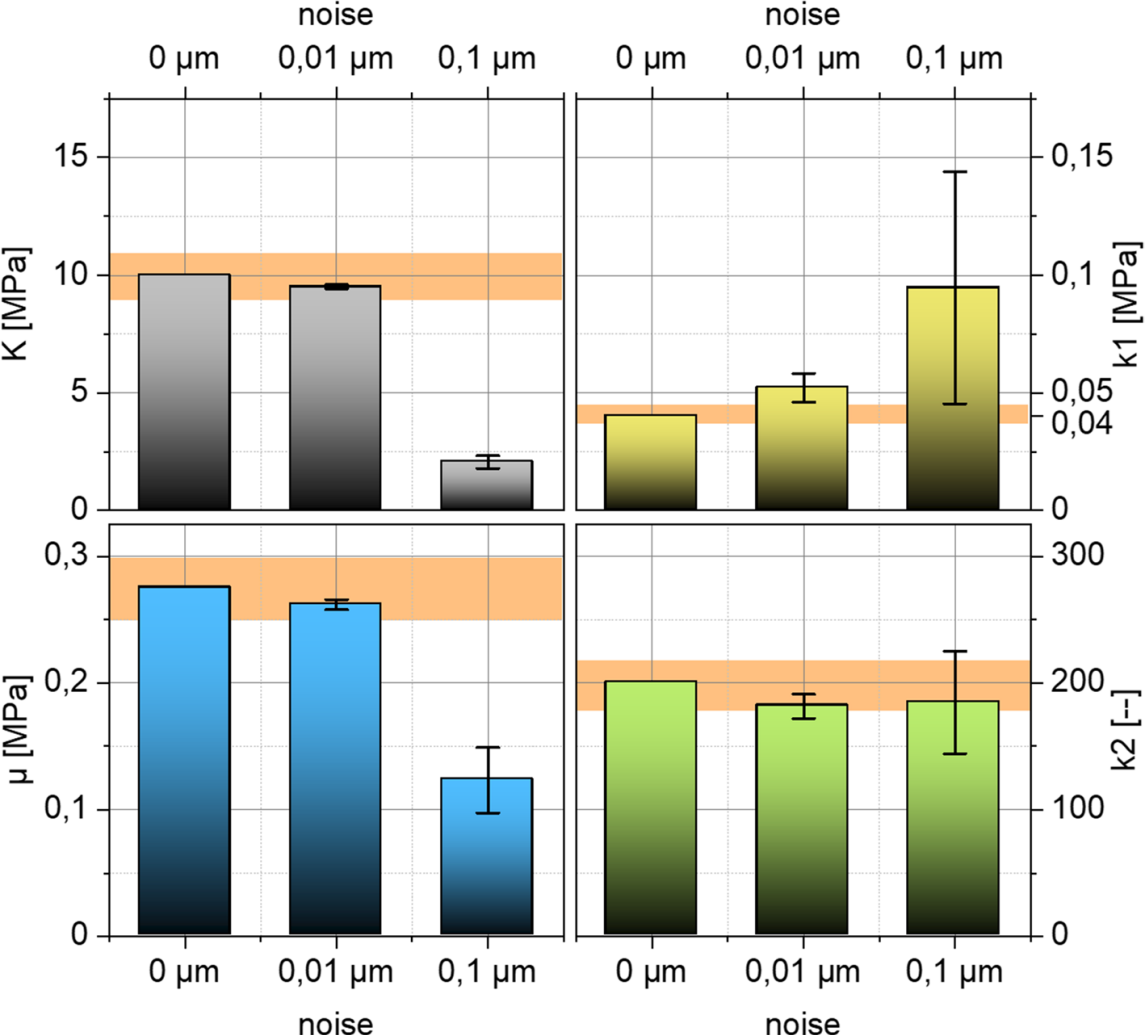
Inversely identified material parameters from the healthy material set as a function of the applied random noise. The diagram contains the ± 10% deviation band to the reference value (orange) as well as the twofold standard deviations determined from 10 repetitions as error bars

The previous investigations are based on the use of the unperturbed full-field displacement associated with the reference data. However, the advised NCT devices measure the displacement data optically and optical systems are subject to numerous environmental influences that cause image noise. In order to investigate the influence of noisy deformation fields on the inverse parameter identification, a superposition of absolute noise on the full-field displacement of the healthy reference data set (H) is considered. For this purpose, a random offset between $-0.01 \mathrm{\mu m}$ and $0.01 \mathrm{\mu m}$ or between $-0.1 \mathrm{\mu m}$ and $0.1 \mathrm{\mu m}$ is added to each component of the displacement matrix $\widehat{{\varvec{u}}}$. For comparison, the nodal displacements in the corneal region have average values of approximately $80 \mathrm{\mu m}$. For random calculation, the RAND function implemented in ANSYS® coupled with the system time is applied. A regularization of the generated noisy displacement matrix $\widehat{{\varvec{u}}}$ in terms of smoothening is intentionally not performed. Figure 8 compares the results of the inverse parameter identification in the presence of noise. As can be observed, the results are clearly unstable as soon as absolute noise of $0.1 \,\mathrm{\upmu m}$ is taken into account.

However, this observation should be put into perspective. Keep in mind that here we considered a rather unrealistically intense noise on purpose in order to challenge the method. The considered noise is random, not Gaussian distributed as may be expected in reality. Furthermore, for the real application unsmoothed, raw pixel data would not be directly considered as geometry input and rather a smoothened surface line would be constructed. In addition to that, the considered noise is completely random in orientation. Considering the 8 nodes of an element, the noise can lead to expansion or contraction of the element volume. The probability that an element contracts or expands because of the noise is thereby many times higher than that the element volume remains constant, since constancy requires all 8 nodes to be shifted by the same offset in the same direction. The violation of incompressibility caused by the noise leads to the underestimation of the penalty parameter $K$. So, the equilibrium between the components of the strain energy function shifts. As a result, the material parameters must compensate the volume change in addition to the deformation and therefore deviate from the target values. This effect increases with the increase in the noise intensity. Interpretation of the noise intensity applied here is rather difficult. Surely, the limitations of the Corvis® ST regarding the resolution of about $15 \mathrm{\mu m}/\mathrm{px}$ ($564 \mathrm{px}$ (Koprowski et al. 2015) to about $8.5 \mathrm{mm}$ (Ambrósio et al. 2013; Reznicek et al. 2013)) are non-negligible. However, it may be fair to state that in principle the use of the raw data from the Corvis® ST should not be directly used and rather smoothened data should be considered instead. That way also incompressible deformations could be enforced in the input data. One strong limitation resulting from the current state of the art regarding technical possibilities should not be omitted here: Although current developments are promising with respect to providing technical solutions for the full 3D measurement of displacements, these full-field data are not yet measurable in the eye during NCT.

### Analysis of morphing approach for the approximation of full-field displacements

**Fig. 9:**
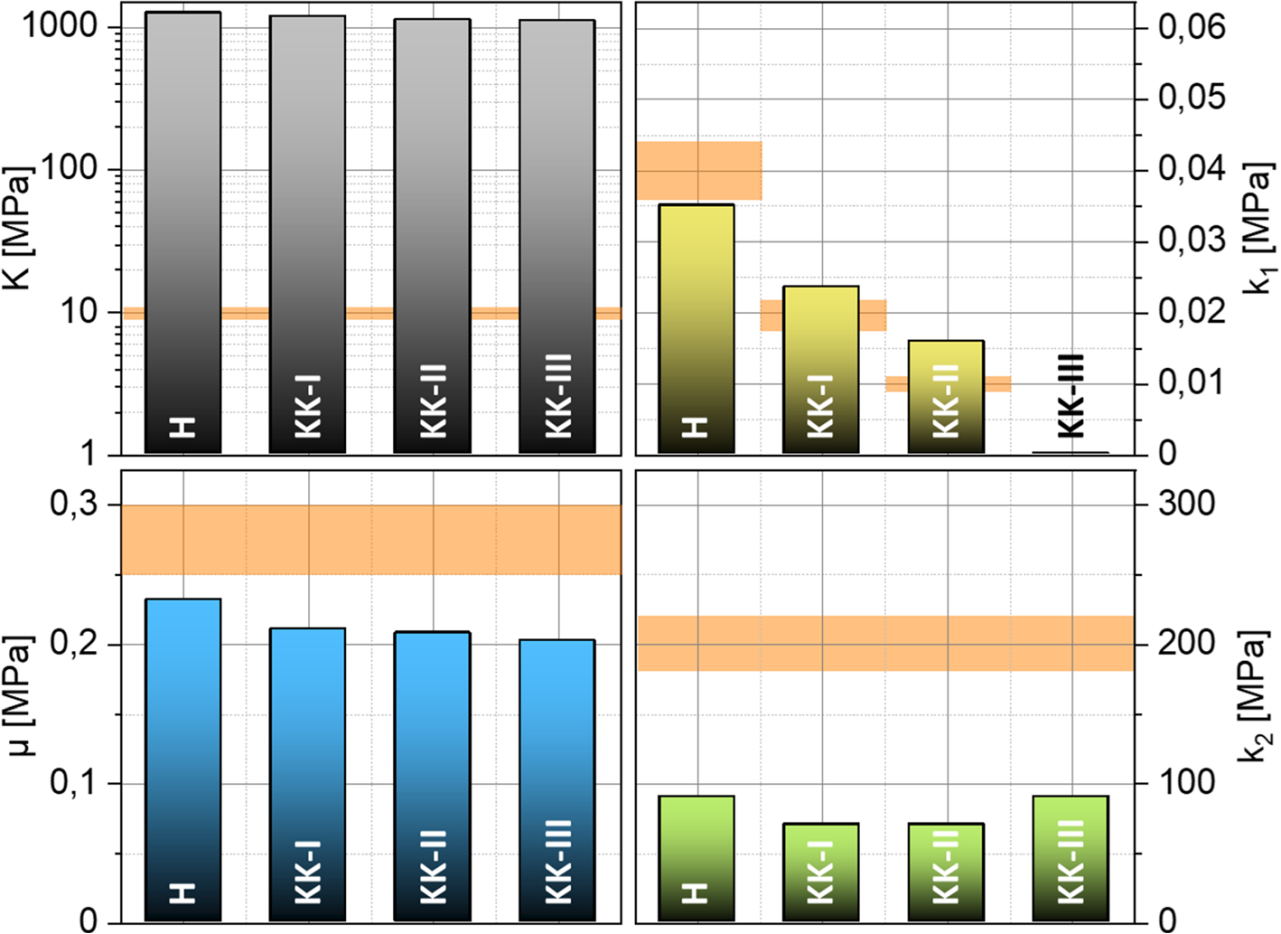
inverse determined material parameters for the four test cases H, KK-I, KK-II and KK-III as well as the ± 10% deviation band to the reference value (orange)

The necessary input data of a full displacement field for the presented approach are, as mentioned above, not detectable by the Corvis® ST today. Accordingly, a mechanical morphing approach for the data enrichment was presented, which serves as a link between possible measurement data and the presented inverse identification apparatus. In the following, it will be shown that this approximative full field contains all necessary information to identify the material parameters of the more complex material model according to Sánchez, Moutsouris and Pandolfi. For this analysis, the reference data sets consisting of virtually measured displacement fields have to be reduced to data currently available during NCT. This means to the coordinates of the 2D deformation contours in the intersection from anterior and posterior corneal surface with a symmetry plane of the eye. Figure 9 presents graphically the results of the parameter identification combined with the mechanical morphing approach for all 4 reduced reference data sets.

As can be seen, the linear–elastic material model in the approach for estimating an equivalent full field provokes the incompressibility much stronger than the penalty parameter of $K=10 \,\mathrm{MPa}$ given in the reference data set. Since the incompressibility is already ensured with $K=10 \,\mathrm{MPa}$, higher values do not have much influence on the results of the material parameters. The parameter $\mu$ shows a slightly increasing deviation with increasing degradation. The average of the deviation is about  − 25 %. The determined $\mu$ is generally smaller than the expected value of $0.275 \,\mathrm{MPa}$. The dimensionless material parameter ${k}_{2}$ is underestimated for all four material data sets. Its relative deviation is about − 60 %. The determined parameter is constant, as specified in the reference data. The relative deviation of the fiber stiffness parameter ${k}_{1}$ increases with increasing degradation, as observed for the parameter $\mu$. On average, the deviation is about 30 %. However, the major result here is that despite the overall only moderate accuracy to determine the precise values of the parameters, a range of reduced collagen stiffness was clearly detectable through the identified values of the parameter ${k}_{1}$. Remember that only cornea surface data of one two-dimensional cross section has been used as virtual input data, just as it would be available from real NCT data. Therefore, this may render the proposed approach feasible as diagnostic tool based on NCT measurements. What remains is to study whether a characteristic eye model as considered here can indeed be used to identify medically relevant ranges of parameter values for a variety of different eyes or whether patient-specific models need to be constructed. This could be addressed in future research where varying eye geometries are studied and clinically compared with real scenarios.

## Conclusions

The authors proposed a method that enables the in vivo identification of structural material properties of the human cornea while being update-free and, thus, furthermore enabling a unique parameter identification in almost real time. The method is based on the EGM and the approach for the identification of nonlinear parameters in Perotti et al. (2017), combined with a new approach to construct approximate full-field kinematics from standard NCT measurements. The functionality of the method was demonstrated on synthetic data of NCT measurements serving as reference.

A finite element model of the human eye was used to synthetically generate full-field displacement from 4 material parameter data sets with keratoconus-like degradations. Thus, the authors generated reference data sets consisting of virtually measured displacement fields related to prescribed material parameters. This allowed to quantitatively demonstrate the accuracy and potential of the proposed approach of EGM combined with the approach for the identification of nonlinear parameters. By having chosen representative synthetic data rather than patient-specific data, the found conclusions were of more general nature in the sense of a feasibility study. In a further step, random absolute noise was added to the displacement fields to investigate the sensitivity to noise. To close the current gap between recordable data and available real-time inverse approach, we proposed and tested an approach for the construction of approximative displacement fields just based on data currently available during NCT, which automatically contains to some extent data smoothing. The authors then quantified the accuracy of the whole method based on the EGM, the nonlinear parameter identification approach and the mechanical morphing approach.

The inversely identified material parameters based on the known full fields have shown the high accuracy of the approach. In this context, it should be pointed out that there exists currently no technical equipment able to measure the required full fields during air pulse tonometry. This point is supported by the performed noise analysis. It has been shown that the approach is sensitive to random noise for already small amplitudes of $0.1\mathrm{ \mu m}$. With an identification time of about $0.1\mathrm{ \mu m}$, the approach is much faster than inverse problems solved by typical forward approaches. These would require many thousands of forward simulations of the NCT where each of those would already require more than 11 h when using equivalent computing resources. The results of the identification based on the data currently available during the NCT and using the mechanical morphing approach showed that a degradation of the parameter ${k}_{1}$ is detectable. This parameter is related to the stiffness of collagen fibers. The deviations in the parameter identification of about $25\,\mathrm{ \%}$ to $30\,\mathrm{ \%}$ for $\mu$ and ${k}_{1}$ and of about $60\,\mathrm{ \%}$ for ${k}_{2}$ are too large for subclinical diagnosis. However, analysis show that the accuracy is sufficient for the identification of diseased tissue properties.

An open issue is still the lack of technical equipment to accurately measure the required full-field displacement during the very fast NCT examination. The mechanical morphing approach to overcome this lack was based on a strong simplification, namely linear elasticity. This was motivated by the fact that initially there is no information regarding the material properties, and thus, it should be avoided to include too much a priori knowledge. However, further improvements may be achieved by extending accuracy here. Furthermore, our approach assumes knowledge about the stress-free geometry of the eye. Currently, this is not measurable. The procedure of Pandolfi and Holzapfel (2008) would be one possible approach to obtain the stress-free geometry. However, it is based on time-consuming forward simulations, which take about $5\,\mathrm{ h}$ for the presented model. Therefore, new methods and approaches must be investigated in the future.

Finally, the authors highlighted that the proposed framework is able to identify the material properties of human eye tissues in vivo and contactless based on constructed full-field displacement. During the NCT, the patient's eye is not injured and there is no need to obtain specimens that could be altered by the removal or environmental conditions. Based on data gathered during NCT, our approach enabled the unique identification of degenerated fiber stiffness. Thereby, it represents a promising new method to be used as additional medical diagnostic tool.

As an outlook, clinical validation is required. For this purpose, series of experiments performed on healthy and diseased eyes could be studied where computer tomography, NCT examination, mechanical testing and pathological analysis are combined as a data set to which the results of our approach can be compared. Thereby, it could be analyzed whether indeed parameter ranges which may be identified by our method correlate with diseased tissues.

## Appendix A

The formulas for the compressive and shear stresses acting on the surface on the cornea, which result from the air puff, are the surface functions Eqs. (15) and (16), as already published in Muench et al. (2019):

15 $$\begin{aligned} p\left( {x,y} \right) = & A1 \cdot \exp^{{ - \mathrm{abs}\left( {C1 \cdot x^{2} + D1 \cdot y^{2} } \right)}} \\ & - A2 \cdot {\text{exp}}^{{ - B2 \cdot {\text{abs}}(C1 \cdot x^{2} + D1 \cdot y^{2} + E_{{\text{c}}}^{2} \cdot \sqrt {C1 \cdot x^{2} + D1 \cdot y^{2} } }} \\ & + A3 \cdot {\text{exp}}^{{ - B3 \cdot {\text{abs}}\left( {C3 \cdot x^{2} + D3 \cdot \left( {y - y_{{\text{c}}} } \right)^{2} } \right)}} + A3 \cdot {\text{exp}}^{{ - B3 \cdot {\text{abs}}\left( {C3 \cdot x^{2} + D3 \cdot \left( {y + y_{{\text{c}}} } \right)^{2}} \right)}} \\ \end{aligned}$$

16 $$\begin{aligned} s\left( {x,y} \right) = & \,A1 \cdot {\text{exp}}^{{ - \left( {C1 \cdot {\text{abs}}(x)^{E1} + D1 \cdot {\text{abs}}(y)^{E1} } \right)}} \\ & + A2 \cdot {\text{exp}}^{{ - {\text{abs}}\left( {C2 \cdot x^{2} + D2 \cdot y^{2} + E_{{\text{c}}}^{2} - 2E_{c} \cdot \sqrt {C2 \cdot x^{2} + D1 \cdot y^{2} } } \right)}} \\ & - A1 \cdot {\text{exp}}^{{ - {\text{abs}}\left( {C3 \cdot x^{2} + D3 \cdot y^{2}} \right)}} \\ \end{aligned}$$

These depend on the one hand on the position (*x*, *y*) but also on the deformation state of the cornea, since the deformation of the cornea naturally has an influence on the flow of the air pulse and thus on the load distribution on the cornea. In order to avoid the time-consuming fluid structure interaction simulation, the authors therefore decided to map this dependence via the parameters $A1 - E1$. These parameters are selected from a lookup table depending on the deformation case, which is quantified by the deformation amplitude *DA* of the cornea. Tables

**Table 5:** Coefficients of the pressure load describing function Eq. (15) as published in (Muench et al. 2019)

Coeff.	Cornea deformation states											
	Unit	1	2	3	4	5	6	7	8	9	10	11
*DA*	mm^2^	0	0.11	0.32	0.54	0.82	1.28	1.61	2.04	2.51	2.94	3.38
*R*	%	86.7	87.0	88.9	89.1	90.3	91.5	92.3	92.8	93.5	93.8	94.5
*A*1	Pa	25 300										
*C*1	${\mathrm{mm}}^{-2}$	0.68	0.68	0.65	0.62	0.59	0.55	0.51	0.45	0.44	0.42	0.38
*D*1	${\mathrm{mm}}^{-2}$	0.89	0.90	0.87	0.85	0.79	0.72	0.66	0.63	0.61	0.59	0.52
*A*2	Pa	0	0	636	874	1756	2553	3137	3024	3137	3485	3853
*B*2	–	0	0	18.3	6.23	7.41	8.86	9.18	6.18	7.94	8.85	8.55
${E}_{c}$	mm	0	0	2.13	2.05	1.91	1.84	1.75	1.75	1.82	1.86	1.74
*A*3	Pa	5219	5234	5120	5111	5095	4976	5003	5002	4958	4914	4845
*B*3	–	1.43	1.45	1.96	2.32	2.23	1.20	0.98	0.56	0.48	0.40	0.29
$C3\cdot {10}^{4}$	${\mathrm{mm}}^{-2}$	1.58	7.47	6.89	7.49	10.5	23.8	38.1	86.8	131	244	333
*D*3	${\mathrm{mm}}^{-2}$	0.71	0.69	0.50	0.43	0.44	0.82	1.03	1.81	2.16	2.64	3.73
${y}_{c}$	mm	4.7										

^5^ and

**Table 6:** Coefficients of the shear stress describing function Eq. (16) as published in (Muench et al. 2019)

Coeff.	Cornea deformation states											
	Unit	1	2	3	4	5	6	7	8	9	10	11
*DA*	mm^2^	0	0.11	0.32	0.54	0.82	1.28	1.61	2.04	2.51	2.94	3.38
*R*	%	95.0	95.1	95.1	95.2	95.1	95.2	95.4	95.7	95.9	96.2	96.2
*A*1	Pa	89.6	64.3	131	140	144	241	260	172	175	185	150
$C1\cdot {10}^{3}$	${\mathrm{mm}}^{-E1}$	11.4	5.4	15.2	18.4	17.6	21.8	28.0	12.8	12.9	17.3	13.4
$D1\cdot {10}^{3}$	${\mathrm{mm}}^{-E1}$	22.4	12.3	35.4	41.0	38.0	48.7	59.1	30.6	30.4	37.7	31.1
*E*1	–	2.23	2.44	2.30	2.21	2.26	2.39	2.29	2.56	2.59	2.46	2.52
*A*2	Pa	169	192	145	143	145	85	111	135	136	140	159
*C*2	${\mathrm{mm}}^{-2}$	0.25	0.19	0.22	0.25	0.31	1.58	0.49	0.80	1.16	1.51	1.06
*D*2	${\mathrm{mm}}^{-2}$	0.44	0.34	0.36	0.39	0.50	1.81	0.52	1.03	1.47	1.84	1.31
${E}_{c}$	mm	1.12	0.90	0.89	0.93	1.10	2.50	1.47	1.90	2.45	2.84	2.41
*C*3	${\mathrm{mm}}^{-2}$	2.41	1.19	0.91	0.83	0.83	1.00	0.87	1.06	1.75	1.79	2.47
*D*3	${\mathrm{mm}}^{-2}$	2.41	1.26	1.23	1.11	1.05	1.45	1.30	1.35	2.66	2.90	4.61

^6^ summarize the parameters for 11 deformation cases.
